# Zinc, Vitamin D and Vitamin C: Perspectives for COVID-19 With a Focus on Physical Tissue Barrier Integrity

**DOI:** 10.3389/fnut.2020.606398

**Published:** 2020-12-07

**Authors:** José João Name, Ana Carolina Remondi Souza, Andrea Rodrigues Vasconcelos, Pietra Sacramento Prado, Carolina Parga Martins Pereira

**Affiliations:** ^1^Kilyos Consultoria, Assessoria, Cursos e Palestras, São Paulo, Brazil; ^2^Department of Pharmacology, Institute of Biomedical Sciences, University of São Paulo, São Paulo, Brazil

**Keywords:** vitamin C, vitamin D, zinc, COVID-19, junctional complex, immunity, nutrients

## Abstract

Some nutrients play key roles in maintaining the integrity and function of the immune system, presenting synergistic actions in steps determinant for the immune response. Among these elements, zinc and vitamins C and D stand out for having immunomodulatory functions and for playing roles in preserving physical tissue barriers. Considering the COVID-19 pandemic, nutrients that can optimize the immune system to prevent or lower the risk of severe progression and prognosis of this viral infection become relevant. Thus, the present review aims to provide a comprehensive overview of the roles of zinc and vitamins C and D in the immune response to viral infections, focusing on the synergistic action of these nutrients in the maintenance of physical tissue barriers, such as the skin and mucous membranes. The evidence found in the literature shows that deficiency of one or more of these three elements compromises the immune response, making an individual more vulnerable to viral infections and to a worse disease prognosis. Thus, during the COVID-19 pandemic, the adequate intake of zinc and vitamins C and D may represent a promising pharmacological tool due to the high demand for these nutrients in the case of contact with the virus and onset of the inflammatory process. Ongoing clinical trials will help to clarify the role of these nutrients for COVID-19 management.

## Introduction

The coronavirus disease 2019 (COVID-19) pandemic highlights the importance of the use of essential nutrients, especially those with immunomodulatory effects that support an organism's natural immune defenses in the event of that or other viral infections (1–3).

Viruses classified as coronavirus belong to the family Coronaviridae, a name derived from the crown-shaped spikes on the pathogen surface, and are characterized by compromising the human respiratory system (4, 5). At the end of 2019, individuals in the city of Wuhan, China, were diagnosed with pneumonia caused by a new coronavirus: severe acute respiratory syndrome coronavirus 2 (SARS-CoV-2) (4, 6, 7). With the rapid increase in the number of positive SARS-CoV-2 infections in all regions of the world, the World Health Organization (WHO) declared COVID-19 a pandemic in March 2020.

The clinical manifestations of this disease have a broad spectrum, including asymptomatic infection, mild upper respiratory tract infection and severe pneumonia with respiratory failure, for which hospitalization with sub-intensive or intensive care is required (8, 9).

Nutrition is a determinant factor for the maintenance of homeostasis and the health of different organs and physiological systems of an organism, including immune function (10, 11). In the current context of the COVID-19 pandemic, the “nutritional status-immune response” dyad of an individual becomes even more significant because in the absence of a widely available vaccine or treatment supported by high-quality evidence, the main therapeutic/preventive measure for the disease lies in the individual response to the virus (2, 3, 12–16). Inadequate nutrition is considered a contributing factor to the emergence of viral infection due to its contribution to weakness of immune system, which increases the rate of infections and the risk of mortality and morbidity. Moreover, viral infections increase the demand for several micronutrients such as vitamin A, B, C, D, zinc, and selenium (17–20).

Dietary supplementation of micronutrients with recognized roles in immune function can optimize the modulation of the body's immune response, reducing the risk of infections (19, 21–25). In this context, zinc and vitamins C and D are the micronutrients for which there is robust evidence of their immunomodulating activity, such that their deficiency, even if marginal, can compromise metabolism and, consequently, their action on the immune system, a concept grounded by the triage theory^1^ proposed by Ames (26) and the Law of the Minimum^2^ proposed by Justus von Liebig in 1840 (10, 17, 24–30).

Considering the importance of adequate levels of nutrients for immune system activity, this review article presents, in a narrative form, a review of the main medical-scientific findings for the relationship between zinc, vitamin C and vitamin D and viral infections, in particular those caused by SARS-CoV-2, demonstrating a confluence of mechanisms in various immune functions, with an emphasis on the integrity of physical tissue barriers composed of epithelial cells and intercellular functional complexes.

## Nutrients and Viral Infections

### Zinc

The immunomodulatory and antiviral activities of zinc have made this mineral and its ionophores candidates against COVID-19 (31, 32). Zinc is essential for the integrity of the immune system (33), with an important role in the maintenance, development and activation of cells during innate and adaptive immune responses. It also plays a role in the integrity of epithelial barriers, which are essential for organism defense and prevention of pathogen entry (34–37). Zinc can modulate the development and activity of T cells, hence reducing the cytokine storm, characterized by high levels of pro-inflammatory cytokines and chemokines that lead to systemic immune response impairment, resulting in acute respiratory distress syndrome (ARDS) or in multiple organ failure (38–40). Zinc deficiency decreases the activity of natural killer (NK) cells and cytolytic T cells, both of which are involved in the destruction of viruses, bacteria and tumor cells (41, 42).

Another important function of zinc is its direct antiviral activity, which makes it essential for the immune response upon viral infection. Increased intracellular concentration of this mineral can reduce the replication of a variety of RNA viruses (43–48) and interfere with the viral proteolytic processing of polyproteins (49). Importantly, in Vero-E6 cell culture, the incubation with low concentrations of zinc (2 μM) repressed SARS coronavirus (SARS-CoV) replication by inhibiting its RNA polymerase (45). Moreover, zinc can enhance interferon (IFN) cytokine signaling against RNA viruses (50–53) and inhibit the activity of angiotensin-converting enzyme 2 (ACE2), which is critical for SARS-CoV-2 entry into host cells (54, 55).

Zinc deficiency affects approximately one-third of the population worldwide (56) and is considered a global nutritional problem, affecting population groups in both developed and developing countries. According to a WHO report, zinc deficiency is responsible for ~1.4% (0.8 million) of annual deaths and 2.9% of loss of healthy life years (total of 28 million years) around the world (56). It is also considered one of the main causes of morbidity in developing countries (57), and it is estimated that 0.5 million women and children die per year in these countries due to zinc deficiency (58–60). Worldwide, about 16% of lower respiratory tract infections occur due to zinc deficiency (56), suggesting a possible link between this nutritional deficiency and the increased risk of SARS-CoV-2 infection and severe disease progression (61).

According to epidemiological data, most deaths from COVID-19 are concentrated in the elderly with common comorbidities such as hypertension, diabetes or obesity (62–64). In general, this group of individuals has a higher prevalence of zinc deficiency (65–69), given that aging is associated with a progressive decline in zinc status in the body due to several factors, including reduced food intake, decreased nutrient absorption efficiency and use of medications (70, 71). Similarly, obese individuals or those with chronic kidney disease often exhibit zinc deficiency (65–68, 72–74).

Increasing zinc deficiency and particularly reductions in intracellular zinc levels in immune cells are associated with greater difficulty in mobilizing rare zinc reserves in the body of elderly individuals and, consequently, with the progressive dysregulation of immune responses (immunosenescence), resulting in higher susceptibility to infectious diseases (71, 75, 76). In general, elderly individuals with chronic diseases or who are hospitalized have even lower levels of minerals than do healthy elderly individuals (71), which may be responsible for the high incidence of infections and age-related degenerative pathologies (70).

During an infection, an organism can mobilize zinc reserves for priority functions, such as those associated with the immune system, leading to a decrease in zinc levels and, possibly, to the lack of zinc to other less essential functions, such as the maintenance of smell and taste, senses often affected in patients with COVID-19. This notion agrees with the triage theory cited above (26).

Zinc also plays an important role in intercellular junction proteins, structures that promote adhesion between epithelial cells and that are necessary for epithelial tissue structure and its function as a selective barrier (34–36). Under zinc deficiency conditions, disruption of intercellular junctions occurs, with a consequent reduction in tissue integrity and impairment of the control of paracellular permeability, allowing the passage of pathogens (34, 36, 77). Conversely, zinc supplementation improves the function of these junctions (34). Considering the important role of zinc in maintaining the integrity of the physical barriers of the skin and mucous membranes against the invasion of pathogens (37), it may also play a role in reducing the risk of contamination by SARS-CoV-2.

The intestinal mucosa is an important cellular barrier that prevents the entry of pathogens and is compromised after SARS-CoV-2 infection. Data from Hubei Province, China, shows that up to 79% of infected patients may present gastrointestinal symptoms, such as diarrhea, vomiting, abdominal pain, and gastrointestinal bleeding (78). The literature reports the occurrence of diarrhea in 2 to 50% of COVID-19 cases (78, 79). In some patients, it was the only symptom presented, which was associated with worse disease prognosis (80, 81) and can reduce zinc levels in the body due to malabsorption and loss during dehydration (82).

Supplementation with zinc was shown to be effective in the treatment of acute diarrhea, which may be due to viral infection (83). That approach is recommended by the WHO for the treatment of children with acute diarrhea to reduce its duration and severity in addition to the risk of new episodes in the following 2 to 3 months (84). Regarding zinc sources, it was found that supplementation with zinc amino acid chelate, compared with placebo and zinc sulfate, had a better effect in reducing the incidence of diarrhea and acute respiratory infection, in addition to resulting in a lower incidence of side effects, in preschool children (22). Zinc reduces the risk of diarrhea through its function in the intestinal barrier (77) and through several mechanisms that act directly on pathogens, including a reduction in the expression of virulence factors (85, 86).

Zinc-deficient individuals are prone to increased respiratory and diarrheal morbidities (87, 88). Furthermore, it was found that zinc supplementation in children with zinc deficiency may reduce the morbidity and mortality related to lower respiratory tract infections caused by the measles virus (89). Zinc administration is also associated with a 41% reduction in the prevalence of childhood pneumonia (23), a lower respiratory tract infection.

Clinical studies have shown that zinc supplementation can also reduce, by up to 54%, the severity and duration of various cold symptoms, such as fever, cough, sore throat, muscle pain and nasal congestion (90–92), which may also occur after SARS-CoV-2 infection. In a randomized double-blind study, 48 volunteers with colds received zinc acetate lozenges (80 mg of elemental Zn/day) supplementation or placebo within 24 h after the onset of symptoms. In comparison to placebo, zinc administration was associated with a significant reduction in the duration of cold symptoms and the total severity score of all symptoms (*p* < 0.002) (90).

In a case report series of four patients with COVID-19, administration of high doses of oral zinc (up to 207 mg/day) was possibly associated with improved oxygenation and fast resolution of shortness of breath after 1 day of treatment. No adverse effects were reported (93). Conversely, a prospective study with 242 patients did not find a significant correlation between zinc supplementation and reduced COVID-19-related mortality (RR = 0.66; 95% CI: 0, 41-1.07; *p* = 0.09) (94). In view of some limitations of the study (single-center retrospective design, possible presence of confounding variables, sample size and a higher proportion of patients treated with zinc), the authors highlighted the need for randomized clinical trials to investigate the potential of zinc in COVID-19 therapy (94).

By the time this review was completed, more than 10 clinical trials involving oral zinc as monotherapy or in association with other compounds have been registered at clinicaltrials.gov and have alrealy started subjects' enrollment (NCT04468139, NCT04472585, NCT04342728, NCT04446104, NCT04335084, NCT04370782, NCT04447534, NCT04326725, NCT04334512, NCT04412746), two of which have already been completed (NCT04485169, NCT04491994). The results of these studies will be important to validate the usefulness of zinc as an adjuvant therapy in COVID-19.

### Vitamin C

Ascorbic acid is a water-soluble micronutrient with antioxidant properties that plays a crucial role in the immune system, supporting the epithelial barrier against the entry of pathogens and the cellular functions of the innate and adaptive immune systems (19, 95).

As an antioxidant, vitamin C prevents damage to biomolecules (nucleic acids, proteins, lipids and carbohydrates) resulting from exposure to oxidants generated by normal metabolism and exposure to pollutants and toxins (96). Furthermore, this vitamin is a cofactor of several enzymes that are involved in the stabilization of the collagen tertiary structure (97), in the biosynthesis of hormones such as norepinephrine, catecholamines and vasopressin (98), and in the methylation of DNA and histones and is therefore important for the occurrence of epigenetic events (99).

Vitamin C levels in the body may vary due to environmental conditions, such as air pollution, and the presence of pathologies, such as type 2 diabetes (95). The elderly population is particularly affected by vitamin C deficiency because chronic or acute diseases are prevalent in this group, and aging is related to reduced vitamin C levels (100–103). For example, low levels of vitamin C (≤ 17 μmol/L) in a population of British elderly individuals were associated with all causes of mortality, including cardiovascular causes (104). In addition, hospitalized elderly patients with acute respiratory infections, when supplemented with 200 mg/day of vitamin C, showed reduced disease severity indices compared to the placebo group (105). Recently, Arvinte et al. (106) conducted a pilot study that included 21 critical COVID-19 patients and observed low serum levels of vitamin C and vitamin D among the patients. In addition, older age and low vitamin C levels appeared to be co-dependent risk factors for mortality, suggesting that serum vitamin C levels contributed to the significance of age as a predictor of mortality (106).

A meta-analysis of 44 studies that used doses of vitamin C starting at 200 mg per day reported a reduction in the duration of the common cold in adults and children, which has been related to the role of this vitamin in supporting the immune system and in reducing the severity of symptoms (107) and is associated with its antihistaminic properties (108). Johnston et al. (21) evaluated the effect of vitamin C supplementation (1 g for 8 weeks) on the symptoms of respiratory tract infections in men with hypovitaminosis C (≤ 45 μmol/L). Although not statistically significant, supplementation reduced the episodes of cold and shortened the duration of infection by 59% compared to the placebo group (−3.2 days; 95% CI: −7.0–0.6; *p* = 0.06) (21).

With respect to acute respiratory infections, it was found that the administration of vitamin C reduced the score for respiratory symptoms of pneumonia in critically ill patients (109). In addition, it was used as an adjuvant in two case reports of patients with ARDS, with an effective reduction in pulmonary edema (110, 111). A high dose of vitamin C in patients with ARDS is related to some beneficial outcomes, such as: reduction of inflammation and organ injuries, decreased pathogen infection and virulence, and optimization of immune defense (112).

In ARDS, liquid and proteins penetrate the alveoli, leading to pulmonary edema. This process occurs due to tight junction damage in the lung endothelium, resulting in increased permeability to fluids, neutrophils and erythrocytes and an excess of these components in the alveolar space (113). The presence of neutrophils in the intravascular and extravascular spaces during acute lung injury is frequently associated with platelets, forming aggregates that, due to their inflammatory thrombogenic activity, lead to inflammatory processes (113).

A study in mice with sepsis and acute pulmonary dysfunction showed that the parenteral infusion of 200 mg/kg vitamin C increased the removal of alveolar fluid, promoted an improvement in the structure and function of the alveolar epithelial barrier, and attenuated the pro-inflammatory response, thus reducing the consequences of sepsis in pulmonary dysfunction (114). These results were obtained by normalization of the constituent proteins of intercellular junctions and by the prevention of rearrangements of cytoskeletons promoted by ascorbic acid (114). In mice deficient in the enzyme responsible for the synthesis of vitamin C (Gulo^−/−^), in a peritoneal inflammatory process, neutrophils were not able to enter into apoptosis and accumulated in the peritoneal cavity due to the absence of recognition and phagocytosis of neutrophils by macrophages, thus reducing the removal of these cells (115). Therefore, vitamin C, as previously mentioned for zinc, plays an important role in the protein components of intercellular junctions, acting in the prevention of the entry of pathogens and in the restructuring of epithelial tissue, in addition to being essential for the removal of neutrophils in damaged tissue, which leads to a reduction in the accumulation of these cells and in the inflammatory process.

In view of the current COVID-19 pandemic, patients with this disease have abnormal chest CT scan results, which reveal bilateral involvement of the lungs with ground-glass opacity, resulting from increased fluid in the lungs, which occurs in 98% of cases. In addition, complications such as ARDS (29%) and secondary infections (10%) may be common (116). Vitamin C may be an adjuvant to ARDS, reduce the deleterious consequences of sepsis associated with acute pulmonary dysfunction, and reduce the incidence of pneumonia by ~80% (117). A meta-analysis of 18 controlled clinical trials showed that oral or intravenous vitamin C reduces both the length of stay in the intensive care unit (ICU) by 7.8–8.6% (*p* ≤ 0.003) and the duration of mechanical ventilation by 18.2% (*p* = 0.001) (118).

Moreover, vitamin C may modulate the cytokine storm (19, 38, 119, 120), characterized by high levels of the pro-inflammatory cytokine interleukin (IL)-6, resulting in increased risk of respiratory failure requiring mechanical ventilation in patients with COVID-19 (121). According to an *in vivo* study with 12 healthy men, pretreatment with vitamin C can reduce the levels of IL-6, released by the vasoconstrictor endothelin-1 (ET-1), thus reducing vascular dysfunction (122). In addition, increased ET-1 expression is also associated with pneumonia, pulmonary hypertension, interstitial pulmonary fibrosis and ARDS (120).

As of the time of completing this review, more than 20 clinical trials with COVID-19 patients are in progress, seven of which have already started enrolling participants, five using vitamin C as an intravenous treatment (NCT04323514, NCT04401150, NCT04357782, NCT04264533, NCT02735707) and two as an oral treatment (NCT04468139, NCT04382040).

In the study by Hiedra et al. (123), 17 COVID-19 patients who needed 30% of oxygen or more received 1 g of vitamin C intravenously for 3 days (123). These patients were receiving hydroxychloroquine, methylprednisolone or tocilizumab as initial treatments. After vitamin C therapy, the levels of some anti-inflammatory markers, such as D-dimer and ferritin, were significantly reduced. However, this study had a small number of participants, had a short duration (only 3 days) and did not investigate the effects of using vitamin C alone. In a case report, a COVID-19 patient underwent intravenous administration of 11 g of vitamin C after developing ARDS and needing mechanical ventilation (124). The high-dose vitamin C treatment was associated with a reduction of both ICU stay and need of mechanical ventilation, as well as a faster recovery of the patient compared to those who did not receive intravenous vitamin C. Importantly, the patient was using the following medications: hydroxychloroquine, azithromycin, colchicine, and zinc sulfate (124).

Despite the limitations of these studies with COVID-19 patients, their results highlight the importance of a more detailed investigation of the use of vitamin C in this disease treatment, since this micronutrient plays a key role in the development, maintenance and expression of the immune response, factors that affect the risk and severity of viral infection, such as SARS-CoV-2.

### Vitamin D

Despite the traditional name, vitamin D is actually a hormone, given that in addition to being endogenously produced, it acts on the regulation of more than 200 genes in different cell types (125–127). Only ~10% of the vitamin D required is obtained from food, its main sources including (i) animals, such as cold deep-water fish, for example, tuna and salmon (D_3_ or cholecalciferol) and (ii) plants, such as edible mushrooms (D_2_ or ergosterol). The remaining 80 to 90% are endogenously synthesized (128), a process that begins in the deep layers of the epidermis, following exposure to ultraviolet (UV) solar radiation, and involves various hydroxylation processes in the liver and kidneys. The final metabolite, 1α,25-dihydroxy-vitamin D [1,25(OH)2 D or calcitriol], is the metabolically active molecule (127, 128).

Variables such as skin pigmentation, aging and topical application of sunscreen may reduce the synthesis of vitamin D by the body (129–131). Importantly, its endogenous production does not lead to toxic accumulation in the organism because there is a system responsible for controlling its levels. During prolonged exposure to UV-B rays, precholecalciferol (previtamin D_3_) is transformed into lumisterol, an inert isomer that maintains the balance of vitamin D_3_ production (132, 133).

In addition to its classic effects on calcium homeostasis and the maintenance of bone health, vitamin D_3_ plays an important role in the body's immune function (17, 19, 134–136). In fact, calcitriol acts as a powerful modulator of the immune system, given that vitamin D (i) has receptors in all immune cells; (ii) is associated with the production of T lymphocytes and the differentiation of B lymphocytes; (iii) optimizes anti-inflammatory functions by altering IL-10 cytokine levels; (iv) induces maturation and differentiation of monocytes and macrophages; (v) is associated with the production of cytokines and chemokines via the nuclear factor-κB (NF-κB); and (vi) induces the secretion of the lysosomal enzymes acid phosphatase and hydrogen peroxide (134, 136, 137). This immunomodulating function of vitamin D is considered complex during viral infections and appears to vary according to the nature of the pathogen and the type of immune function responsible for the resolution of the disease (136, 137).

Besides immune cells, the vitamin D receptor (VDR) is also found in pulmonary epithelial cells. When activated, VDR results in the expression of defensins and catelicidins, peptides with antiviral activity through direct action or via immunological modulation (138–140). It has been speculated that, during vitamin D deficiency, the impaired antiviral immune response in COVID-19 patients may be due to the reduction in LL37 levels, an antimicrobial peptide derived from catelicidin (139).

Vitamin D may also attenuate exacerbated inflammatory responses by downregulating pro-inflammatory cytokines, such as tumor necrosis factor (TNF)-α and IL-6, involved in the development of cytokine storm during COVID-19-related ARDS (136, 140, 141). In fact, preliminary data from more than 5,000 patients with COVID-19 suggested a relationship between vitamin D deficiency and severity of cytokine storm, indicated by high serum levels of the inflammatory marker C-reactive protein (CRP) (142).

A meta-analysis of 25 randomized controlled studies showed that vitamin D_3_ supplementation was associated with a lower risk of developing acute respiratory infections (OR=0.88, 95% CI: 0.81–0.96; *p* < 0.001) and that the positive effect of supplementation was even more pronounced in individuals with vitamin D insufficiency (serum levels below 25 ng/mL) at the beginning of the intervention (143).

In addition to its role in cellular and humoral immunity, vitamin D, similar to zinc and vitamin C, plays an important role in the formation and maintenance of epithelial and endothelial barriers, including lung tissue (17, 19, 144–146). Using a vitamin D receptor knockout mouse model, Chen et al. (146) observed that in the absence of this hormone signaling, the animals exhibited lung inflammation and impaired lung function, which can be explained by the increased pulmonary permeability resulting from impairment of the integrity of the epithelial barrier. The authors demonstrated a reduction in the expression of essential proteins for the maintenance of tight junctions, such as claudins. Thus, in vitamin D deficiency, the lungs lose epithelial integrity, becoming more susceptible to inflammatory processes and pathologies such as asthma, chronic pneumonia and cancer (146).

According to the Endocrine Society, serum levels of 25(OH)D below 20 ng/mL indicate vitamin D deficiency, whereas levels between 21 and 29 ng/mL indicate its insufficiency (147), both of which compromise the immunomodulatory functions of this hormone and have been associated with increased susceptibility to viral and bacterial infections (17, 125, 134).

Some studies suggest that there is a correlation between vitamin D deficiency and susceptibility to SARS-CoV-2 infection and disease severity (24, 106, 140, 148–152). Ilie et al. (149) found a negative correlation between the average serum vitamin D levels in 20 European countries and the COVID-19 cases and mortality per million population. Severely low levels of vitamin D have been identified in the elderly, especially in Switzerland, Italy, and Spain (149).

A meta-analysis of seven retrospective studies reported an average serum vitamin D level of 22.9 nmol/L in 1,368 patients with COVID-19 (140). Significantly lower serum levels of vitamin D were associated with patients with poor disease prognosis compared to those with good outcome, representing a standardized mean difference (SMD) of −5.12 (95% CI: −9.14, −1.10, *p* = 0.012). The difference in vitamin D levels was also substantial between surviving patients and those who died (SMD = −14.6, 95% CI: −15.3, −13.8). The authors concluded that vitamin D deficiency plays an independent causal role in COVID-19 severity and that preventive or therapeutic supplementation in populations at risk can be useful to prevent poor disease outcome (140).

Similarly, a subsequent study with an Israeli cohort of 7,807 subjects identified significantly lower levels of vitamin D among those who tested positive for COVID-19 compared to those who tested negative. Low plasma level of vitamin D (<30 ng/mL) was considered an independent risk factor for COVID-19 infection (OR = 1.58; 95% CI: 1.24–2.01; *p* < 0.001) and hospitalization (OR = 2.09; 95% CI: 1.01–4.30, *p* < 0.05) (150).

As previously mentioned, most deaths from COVID-19 are concentrated in the elderly with comorbidities, and these individuals also have vitamin D deficiency (24). Studies demonstrate the correlation between low levels of calcitriol and pathologies such as cancer, diabetes, hypertension and heart disease (24, 106). Likewise, there is also a known relationship between aging and a reduction in endogenous vitamin D synthesis, which can be explained by the increased levels of parathyroid hormone (PTH) (153). Specifically regarding the elderly population, epidemiological studies indicate that hypovitaminosis D is also associated with increased morbidity and mortality in general (154), whereas a meta-analysis (75,927 participants and 38 studies) showed that vitamin D_3_ supplementation significantly reduced mortality (RR = 0.94; 95% CI: 0.91–0.98; *p* = 0.002) (155).

Given the COVID-19 pandemic, the WHO suggested social isolation as a measure for restricting the spread of the virus. This measure may also contribute to the reduction in serum vitamin D levels because confinement and possible lack of exposure to the sun prevent the endogenous production of this hormone, reducing the body's capacity to fight COVID-19 (156). Alternatives to prevent serum vitamin D deficiency, such as drug supplementation or the ingestion of foods rich in vitamin D, such as fatty fish, cod liver oil and egg yolk, should be considered during this period of isolation (156).

Finally, another important characteristic of the disease is its pathophysiology. SARS-CoV-2 infection starts with the interaction of the virus with the enzyme ACE2, reducing its activity. Importantly, ACE2 has a key role in counteracting ARDS and acute lung injury. Xu et al. (157) demonstrated that calcitriol is able to upregulate ACE2 and downregulate renin and angiotensin II in the lung tissue of rats exposed to lipopolysaccharide as a model of ARDS (157). In this scenario, recent literature have suggested that vitamin D can act by targeting ACE2 down-regulation in SARS-CoV-2 infection, which could be a potential therapeutic approach to COVID-19 and induced ARDS (158–161). However, further studies must be performed to understand the impact of ACE2 modulation for COVID-19 (17, 24, 158–162).

Considering the observations presented, it is suggested that vitamin D deficiency may be the common variable among elderly individuals and patients with underlying diseases, populations more susceptible to complications and mortality by COVID-19. That may justify the use of vitamin D supplementation as a measure of maintenance and expression of the immune response, which are important for reducing the risk and severity of viral infection as well as for mitigating the symptoms of this disease (163–165).

Clinical studies are warranted to determine the effects of vitamin D supplementation in patients with COVID-19. As of the writing of this review, about 30 clinical studies involving oral vitamin D supplementation in COVID-19 patients are registered at clinicaltrials.gov, 12 of those have started enrolling patients (NCT04449718, NCT04487951, NCT04407286, NCT04411446, NCT04502667, NCT04459247, NCT04403932, NCT04335084, NCT04344041, NCT04334512, NCT04386850, NCT04482673) and two are already completed (NCT04407572, NCT04435119).

Next, the formation of the structural maintenance and activity pathways of intercellular junctions whose integrity is an important barrier to the penetration of viruses, bacteria and allergens will be addressed. In this particular context, the nutrients in question participate in metabolic pathways or constitute synergistic or even confluent structures, which reveals their interdependence, both to evidence a loss of selectivity of epithelial barriers in a deficient individual as well as for their repair when supplemented.

## Synergistic Action of Nutrients on Physical Barriers

The outer and inner surfaces of an organism, such as the skin and mucous membranes, are the first line of defense against pathogens, toxins and other foreign bodies, as they form physical barriers that prevent entry (17). SARS-CoV-2 is able to penetrate physical barriers when they are vulnerable. Thus, it can be transmitted directly, when there is physical contact with the infected person, or indirectly, when the infection occurs through droplets (> 5 μm) and aerosols (<5 μm) in suspension or through fomites, where steel and plastic are the materials in which this virus is more viable (166).

Droplets containing SARS-CoV-2 are transmitted when they come in contact with the oral, respiratory tract and intestinal mucosa and the conjunctiva, tissues that express ACE2 necessary for the entry of this virus into the body. However, whether the consumption of food contaminated with SARS-CoV-2 can cause its transmission through the digestive system remains to be confirmed (166–171). Thus, the physical and functional integrity of the physical barriers, especially epithelial cells, is important for reducing the risk of SARS-CoV-2 contagion.

**Figure 1:**
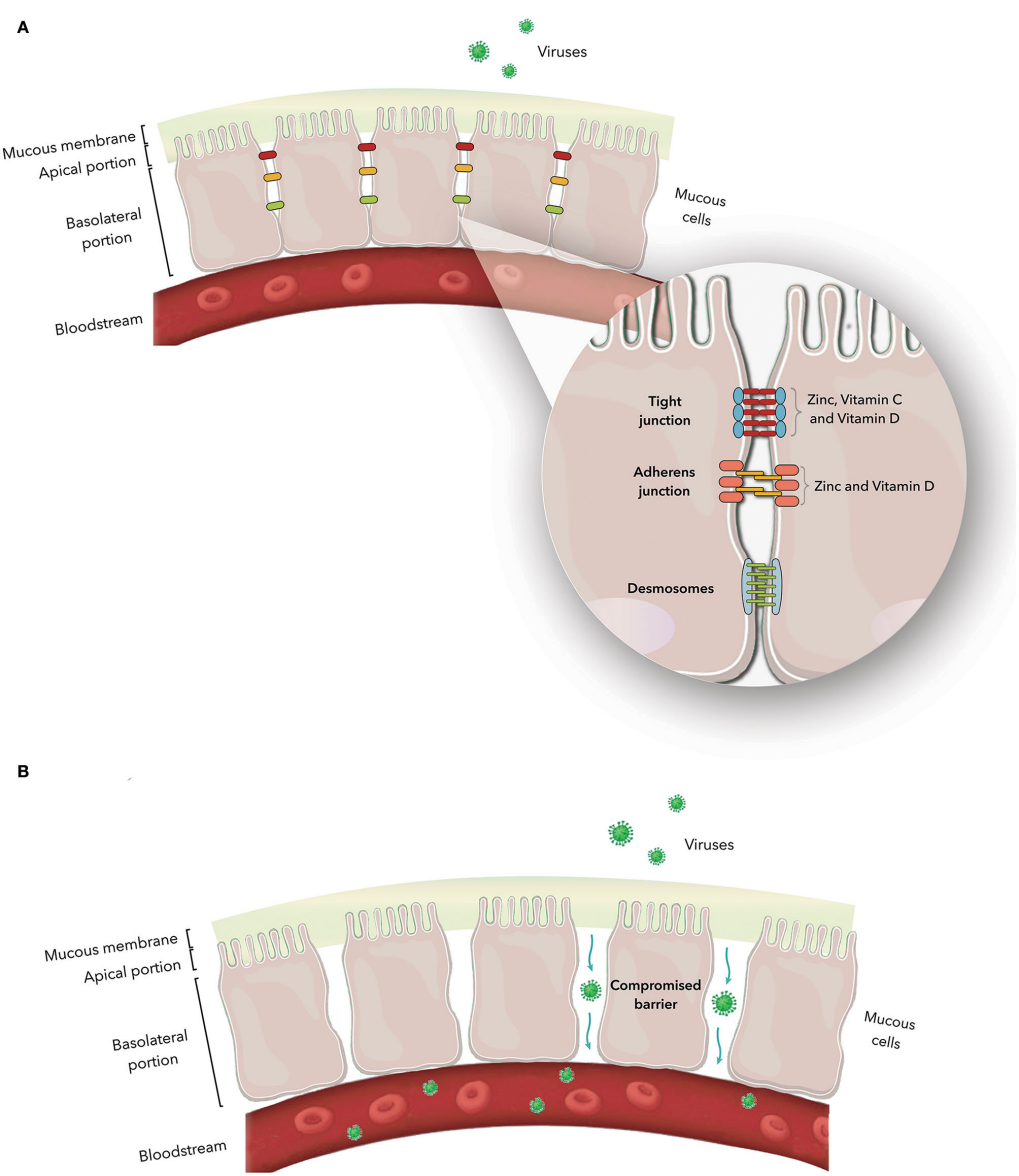
**(A)** Junctional complex in epithelial cells. The magnification shows the arrangement of these structures in the paracellular space and the action of zinc and vitamins C and D on tight and adherens junction proteins. **(B)** Junctional complex dysfunction and its consequences.

The epithelial cells are joined by distinct intercellular junctions, known as the epithelial junctional complex, which is formed by tight junctions, adherens junctions and desmosomes (172, 173) (Figure 1). Because they act as a selective barrier, this complex prevents the entry of pathogens and toxins into the bloodstream.

Nutrient deficiency, the presence of specific pathologies, and interactions with pathogens are some of the possible causes of structural and functional dysfunctions of the junctional complex. Once the junctional complex is compromised, the organism loses its selective barrier and becomes susceptible to the entry of harmful agents, such as viruses, into the bloodstream (114, 172, 174–176). Table 1 describes the constituent proteins of the junctional complex and the functions of each junction.

**Table 1:** Functions and protein constituents of the junctional complex.

**Junctional complex**	**Function**	**Constituent proteins**	**Cytoskeletal adaptor proteins**
Tight junction	Seal intercellular spaces; selective paracellular diffusion	Claudins (26 members of this family); proteins of the MARVEL domain (occludin, tricellulin and MARVELD3)	Zonula occludens proteins (ZO): ZO1, ZO2, ZO3; cingulin; MAGI; PAR3; PAR6 PALS1 PATJ
Adherens junction	Connect the actin filament bundles between the adjacent cells	Classical cadherins (E-cadherin) Nectins	α-catenin, β-catenin, plakoglobin (γ-catenin), catenin p120, vinculin, α-actinin, FFA6
Desmosomes	They connect the intermediate filaments between adjacent cells	Cadherin (desmoglein, desmocollin)	Plakoglobin (y-catenin), plakophilin, desmoplakin

*MAGI, membrane-associated guanylate kinase inverted; PAR, partitioning defective; PALS1, protein associated with Lin-1; PATJ, PALS1-associated tight junction (PATJ); AF6, ALL1-fused gene from chromosome 6 protein; Adapted from (172, 173)*.

Tight junctions are essential for the establishment of the barrier between different compartments of the organism, and their main function is selective paracellular diffusion, which restricts the passage of molecules according to size and ionic charge (172, 173). The selective diffusion promoted by tight junctions is considered a key process for the maintenance of organ and tissue homeostasis (172).

Adherens junctions have an anchoring function, connecting actin filaments between cells and have two subcomplexes: nectin-based adhesions, which are important for the initial attachment between neighboring cells and for the establishment of apical-basolateral polarity, and cadherin-based adhesions, which strengthen intercellular binding. Evidence shows that these two junctions have an interconnection, which can be either physical or through signaling (177).

As with adherens junctions, desmosomes also have anchoring functions, connecting intermediate filaments between cells. This connection ensures the mechanical integrity of the tissues, which is essential for tissues susceptible to mechanical stress, such as skin and heart (178). Desmosomes also interact physically and through signaling pathways with tight and adherens junctions (178). Thus, the junctional complex interacts with each other dynamically to perform its functions effectively.

Some viruses and bacteria, such as the hepatitis C virus, influenza A virus, SARS-CoV, and the bacterium *Helicobacter pylori*, are known to interact with tight junction proteins, thus hindering the integrity and function of these barriers (172, 179, 180). Furthermore, diseases such as chronic inflammatory conditions and cancer can also lead to tight junction dysfunctions, but it is not known whether this dysregulation is the cause or consequence of these pathologies (172).

Zinc and vitamins C and D act in synergy, promoting the integrity and function of some of junctional complex proteins, as will be detailed below.

### Zinc

Zinc is essential for the integrity and homeostasis of the intestinal barrier (181–183). In an experimental colitis model in rats, analysis by electron microscopy showed that zinc supplementation reduced intestinal permeability due to its action on tight junctions (34). In human intestinal carcinoma cells (Caco-2 line) and mouse colon, intracellular depletion of zinc increased the permeability of the intestinal barrier because tight junctions were impaired by a significant reduction in the protein levels of occludin and claudin-3 (176). Supplementation with 100 μM zinc reestablished barrier homeostasis, reducing the permeability of tight junctions (176).

Claudins, the constituent proteins of tight junctions, selectively control the size and charge of molecules that diffuse through the paracellular space. Two types of claudins can be differentiated based on their properties: those that seal the membrane (claudin-1, -3, -4, -5, -6, -8, -12, -18, and -19) and those that form pores (claudin-2 and−15); the latter allow the passage of molecules (184). An *in vitro* study with Caco-2 cells showed that zinc treatment increased transepithelial electrical resistance and mannitol flux, factors that are dependent on the integrity of tight junctions. Based on the results obtained, it was found that claudin-2 and claudin-7 protein levels were significantly reduced after zinc supplementation (77, 185–188). The reduction in these proteins with zinc supplementation influences the increased resistance of the epithelial barrier, which is associated with reduced electrolytic permeability. In turn, the observed increase in mannitol flux, which indicates increased non-electrolyte permeability, is an indication that zinc performs a fine adjustment in the junctional complex, acting differently according to the type of electrolyte (77).

In addition to altering the structure of tight junctions, zinc deficiency also compromised adherens junctions, leading to the delocalization of E-cadherin and β-catenin proteins as well as of the cytoskeleton in Caco-2 cells. This dysfunction in the organization of the intestinal epithelium led to increased permeability and, consequently, neutrophil infiltration in the paracellular space, inducing an inflammatory response (36). Accordingly, in zinc-deficient lung epithelial cells, exposure to cytokines increased cell death by apoptosis and barrier dysfunctions, with E-cadherin and β-catenin proteolysis (189). Apoptosis and barrier dysfunction were directly proportional to the level of intracellular zinc depletion and the time of exposure to depletion associated with acute inflammation (189). Conversely, zinc supplementation reversed the impairment of adherens junctions and was effective in preserving cellular integrity and barrier function (189). Based on these findings, the authors suggested that at the beginning of an inflammatory response in the lung, the mobilization of zinc to epithelial cells is an essential innate response both to increase immune function and to protect other cells from damage caused by inflammation (189). Likewise, zinc-treated Caco-2 cells did not show neutrophil infiltration, indicating that the replenishment of zinc levels restored the integrity of the epithelial barrier and prevented the inflammatory process (36).

### Vitamin C

Vitamin C, like zinc, alters the expression of some tight junction proteins. In a mouse model of induced abdominal peritonitis and subsequent acute lung tissue damage, intraperitoneal administration of 200 mg/kg ascorbic acid and dehydroascorbic acid in a mouse model of induced abdominal peritonitis and subsequent acute lung tissue damage resulted in downregulation of pro-inflammatory chemokines, reduced infiltration of neutrophil polymorphonuclears in the lung and lower severity of tissue damage. In the non-supplemented group, abdominal peritonitis induced in animals led to the loss of alveolar barrier function for small solutes, with a significant increase in the expression of claudin-2 and -4, and reduction in the levels of claudin-18, occludin and the cytoskeletal adaptor protein zonula occludens 1 (ZO1) (114). Supplementation with ascorbic acid significantly reduced pulmonary edema by preserving the epithelial barrier, preventing changes in the expression of these proteins and preserving the paracellular permeability of ions and small molecules, in addition to preventing the rearrangement of the cytoskeleton and of actin (114).

Vitamin C also preserves the integrity of occludins, preventing damage to tight junctions associated with endothelial barrier dysfunction (190, 191). This barrier dysfunction involves the production of the reactive species superoxide anion radical (${\text{O}}_{2}^{\cdot -}$), which reacts with nitric oxide (NO), forming peroxynitrite (ONOO^−^), also with high potential to induce damage to the endothelial barrier and dephosphorylation of occludins. Vitamin C inhibits the enzyme NADPH oxidase, responsible ${\text{O}}_{2}^{\cdot -}$ production, and eliminates ${\text{O}}_{2}^{\cdot -}$ and ONOO^−^, thus avoiding the dephosphorylation of occludins and the consequent loosening of tight junctions (190–192).

### Vitamin D

Vitamin D and its receptor exert an influence on tight junctions, participating in the expression and function of the proteins ZO1, occludin and claudin (19, 175). Fujita et al. (193) found that levels of claudin-2 and claudin-12 were low in the duodenum, jejunum, ileum and colon of vitamin D receptor knockout mice, thus compromising calcium uptake by the intestine (193). In addition, the expression of these two claudins was induced in Caco-2 cells in a dose-dependent and time-dependent manner by treatment with 1α,25-dihydroxy-vitamin D, showing that both are vitamin D signaling targets. Based on these findings, the authors suggest that claudin-2 and claudin-12 form paracellular Ca^2+^ channels in enterocytes and are important for the homeostasis of this cation (193). In human corneal epithelial cells, the metabolites 1,25(OH)2D_3_ and 25(OH)D_3_ increased barrier function, with increased expression of occludin proteins (194).

Other evidence of the function of vitamin D in the integrity of tight and adherens junctions is found in human colon cancer cell lines. Treatment with 1,25(OH)2D_3_ increased the expression of some proteins from these junctions, such as ZO1, ZO2, occludin, E-cadherin and vinculin, leading to decreased membrane permeability, as shown by transepithelial electrical resistance throughout treatment (195). E-cadherin transmembrane protein is important for maintaining the polarized epithelial cell phenotype as well as for adhesion between cells (196, 197). Its importance is revealed by the fact that the loss of E-cadherin expression, a common event during the transition from adenoma to carcinoma, promotes considerable epithelial morphological changes and the acquisition of invasive capacity (198–200). Treatment of colon cancer cells with vitamin D reestablished the compromised tissue morphology due to the increase in the expression of tight and adherens junction proteins.

## Conclusion

Several minerals and vitamins have antioxidant, immunomodulatory and antimicrobial roles which could be helpful for the immune response against the SARS-CoV-2 virus. In the absence of a widely available treatment or a vaccine for COVID-19, supplementation of micronutrients emerges as an important measure to improve the immune system and to prevent the development of severe symptoms. Some of these micronutrients are the vitamins A, B, C, D and E, and minerals such as selenium, magnesium, and zinc (17, 19).

In this review, the role of zinc, vitamin C and vitamin D for immunity was explored since these micronutrients show the strongest evidence for immune support (17). In this scenario, the mentioned studies demonstrate that zinc and vitamins C and D are integral parts of the immune system and show synergistic functions at various stages of the host defenses, such as the maintenance of the integrity of biological barriers and the functionality of cells that make up the innate and adaptive systems. Therefore, the deficiency or insufficiency of these key nutrients, acting in synergy in tight and adherens junction proteins, can lead to impairment of mucosal epithelial cells, possibly making them more susceptible to pathogen entry, such as SARS-CoV-2.

Overall, the medical literature demonstrates that the supplementation with zinc, vitamin C and vitamin D can mitigate viral respiratory infections. Thus, in the context of the COVID-19 pandemic, the supplementation with such nutrients may be characterized as a widely available, safe and low cost measure that can be useful to cope with the increased demand for these nutrients in case of contact with the virus and onset of the immune responses, as well as to lower the risk of severe progression and prognosis of this viral infection.

Ongoing clinical trial will provide more information on their effect on COVID-19 patients.

## Author Contributions

JN contributed to the study concept. JN and CP critically reviewed the article. JN, AS, AV, and CP contributed to the design of the manuscript and figure preparation and edition. JN, AS, AV, PP, and CP contributed to the acquisition and analysis of data and drafted the manuscript. All authors gave final approval for all aspects of the work, agreed to be fully accountable for ensuring the integrity and accuracy of the work, and read and approved the final manuscript.

## Conflict of Interest

The authors declare that the research was conducted in the absence of any commercial or financial relationships that could be construed as a potential conflict of interest.

## Footnotes

^1^The triage theory postulates that in the event of a nutrient deficiency, that nutrient is allocated primarily to functions related to immediate survival while long-term functions are impaired (26).

^2^The Law of the Minimum says that the nutrients that are in an organism in less quantity or deficient become the limiting factor of metabolic reactions even if the other nutrients are present at adequate levels (27–29).
